# Spontaneous Imbibition of Capillaries under the End Effect and Wetting Hysteresis

**DOI:** 10.1021/acsomega.1c06155

**Published:** 2022-01-28

**Authors:** Leilei Zhang, Keliang Wang, Huiming An, Gen Li, Yu Su, Wei Zhang, Xinyi Yang

**Affiliations:** † Key Laboratory of Enhanced Oil and Gas Recovery, Ministry of Education, Northeast Petroleum University, Daqing 163318, China; ‡ Baili College of Petroleum Engineering, Lanzhou City University, Lanzhou 730070, China

## Abstract

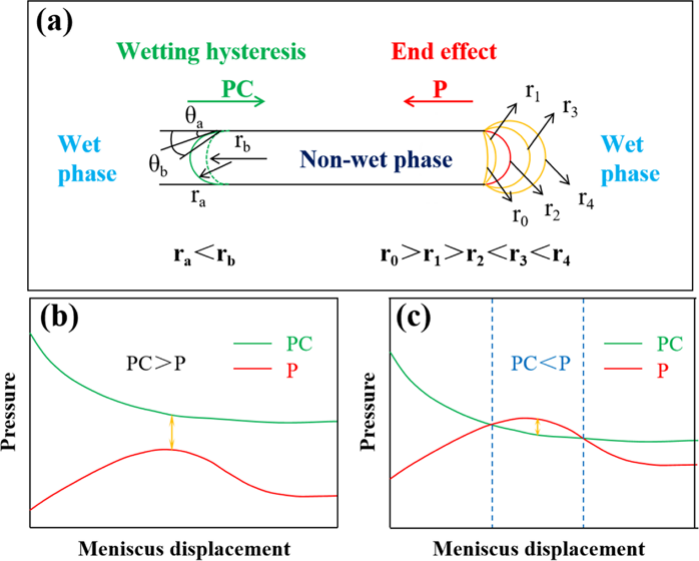

The phenomenon of spontaneous imbibition is widely present in the development process of tight oil/gas reservoirs. To further explore the spontaneous imbibition behavior of capillary tubes to provide theoretical and methodological references for the study of microscopic porous media imbibition phenomena, the capillaries that can be observed with the naked eye on the order of 10–100 μm were selected as research objects. Based on the theory of interface chemistry, the capillary end effect, and wetting hysteresis, the influence of the additional pressures generated by the two-phase interface on the spontaneous absorption of the horizontal capillary was studied. Some of the capillaries were processed for wettability, and then the water wettability of different capillaries was measured by the introduced concept, which is the conversion height of the self-absorption phase in the capillary. The capillaries were horizontally placed in the liquid for a spontaneous imbibition experiment, and the air–liquid two-phase menisci behavior was observed at the same time, and then the influence of water wettability, surfactant, and capillary diameter on spontaneous imbibition was discussed. It was found that in the equal diameter capillaries, the spontaneous air–liquid imbibition behavior of capillary tubes with different water wetting properties is different in sensitivity to surfactants and tube diameters; when surfactants are used to improve capillary water wettability to increase spontaneous imbibition efficiency, the initial water wettability of the capillary and the comprehensive changes in the capillary pressure caused by interfacial tension should be considered.

## 1 Introduction

Capillary spontaneous imbibition refers to the phenomenon that the wet phase displaces the nonwet phase only by capillary pressure without an external force. This phenomenon is widespread in industries such as construction, biochemistry, farmland irrigation, and mineral development.^1−5^ Waterproof and antiseepage of high-rise building roofs,^6^ soilless cultivation of plants by imbibition,^7^ water-saving irrigation using soil imbibition,^8^ development of coalbed methane and tight oil/gas reservoirs,^9^ etc. are all closely related to spontaneous imbibition.

With the development of the human society, the continuous expansion of the economic system, and the continuous increase of energy consumption, oil and natural gas, as important fossil energy sources, play a vital role in the progress of science and technology. Shale gas and tight oil represented by North America have changed the world energy pattern.^10−12^ The newly discovered oil/gas burial conditions in the Asia–Pacific region are becoming increasingly complex and demanding, especially low permeability and tightness.^13−15^ Spontaneous imbibition, as an important mechanism for the development of low-permeability oil/gas reservoirs, has been increasingly attracting attention from petroleum workers.

The behavior of the two-phase menisci in the capillary under different conditions is the basis for studying the influence on spontaneous imbibition, which is related to the matrix pore structure, the matrix opening surface, and reservoir/fluid physical properties in low-permeability oil/gas reservoirs.^16^ Washburn^17^ connected one side of a capillary with water and the other side with air. Under the action of the capillary pressure, water spontaneously enters the capillary and the air is discharged. He found that the displacement of the water–air meniscus is proportional to the square root of the infiltration time. Mason and Morrow^18^ derived a single capillary flow equation suitable for immiscible two phases with different viscosity ratios on this basis. Dong^19^ proposed an ideal double-capillary connection model with different pipe diameters and believed that the pressures in the same phase area of the two capillaries are equal. However, Dong and Ruth^20^ proposed different calculation methods for the meniscus movement speed in the capillary with a larger diameter. Wang^21^ considered the influence of fluid flow between double capillaries and believed that the fluid flow between two capillaries mainly occurs near the oil–water interface. Unsal et al.^22,23^ made irregular nonequal diameter capillary models with different degrees of lateral connectivity to study the curved surface behavior in small and large capillary diameters, and it was found that when the side is disconnected and the nonwet phase end is not closed, the curved surface migration velocity in the large capillary is higher, while when the side is connected or the nonwet phase end is closed, the curved surface migration velocity in the small capillary is higher. Hatiboglu et al.^24,25^ studied the relative flow between fracture and the matrix by a microscopic visualization model and a microscopic filling model. They found that the wet phase preferentially enters the small pore, and the nonwet phase is discharged through the larger pore. At present, the research on the interfacial motion behavior of the multiphase fluid in capillaries is mostly focused on the micrometer scale.^26−28^ During the experiment, a capillary is generally placed horizontally or vertically, the pressure regulating device is established at both ends of the capillary to control the entry of liquid in the capillary and pressure difference, and a microscope with a high-speed camera is used to observe the movement speed and displacement of the two-phase interface. However, the effects of the additional pressure generated by wetting hysteresis and the end effect on the spontaneous imbibition behavior are less studied.

To further explore the spontaneous imbibition behavior of capillary tubes and their influencing factors to provide theoretical and methodological references for the study of microscopic porous media imbibition phenomena, the capillaries that can be observed with the naked eye on the order of 10–100 μm were selected as research objects. The concept of the conversion height of the vertical self-absorption phase of a capillary tube was introduced. Through the measurement of the converted height, the water wetting performance of different capillaries was compared. The capillaries with different water wetting properties were horizontally placed in the liquid, and the initial state that the wetting phase is immersed into one end of the capillary with the help of vibration was created to perform a spontaneous imbibition experiment. By observing the behavior of the two-phase interface, the spontaneous imbibition of the capillary under the dual effects of wetting hysteresis and the capillary end effect was discussed; at the same time, the influence of the water wettability, surfactant, and capillary diameter on the spontaneous absorption of the capillary was analyzed.

## 2 Results and Discussion

Experimental methods and experimental materials are given in Section 4. Schematic diagrams of experiments and the water quality analysis of the experimental water are shown in Section 4.

### 2.1 Capillary Water Wettability Measurement

The capillary pressure is affected by the dual effects of interfacial tension and wetting properties. According to the parameter relationship in the capillary pressure equation (eq 1), for the same diameter capillary, when the two-phase fluid is determined, the larger the value of Δρ × *h*, the stronger the water wettability of the capillary. To determine the water wettability of the capillary by the rising height of the wet phase in the vertical capillary, the conversion height *H* = Δρ × *h* was defined.
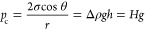
1where σ is the surface tension, *r* is the capillary radius, θ is the contact angle, Δρ is the density difference between the two phases, *h* is the height of the meniscus, and *H* is the converted height of the meniscus.

**Figure 1:**
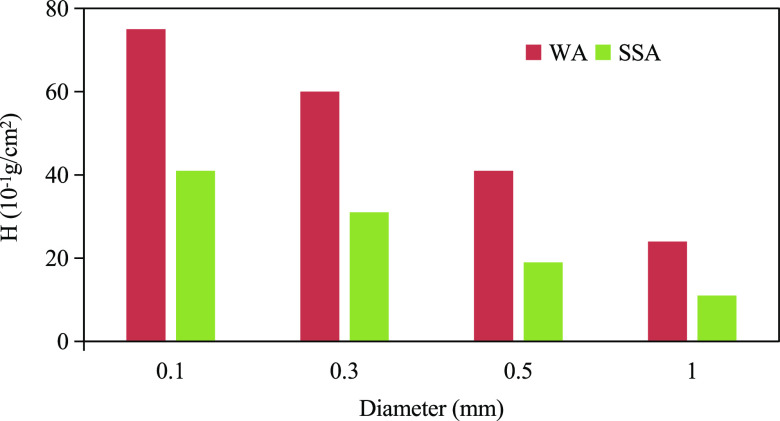
Quartz capillary water wettability.

As shown in Figure 1, under water–air interface conditions (WA), the *H* in the quartz capillary is a positive value, so relative to the air, water is the wet phase. However, under the surfactant solution–air interface conditions (SSA), the value of *H* is reduced and the capillary pressure decreases. As for how much water wettability may be changed after changing from WA to SSA, a comparative discussion will be made in the section that water wettability of quartz capillaries treated with mineral oil was measured.

**Figure 2:**
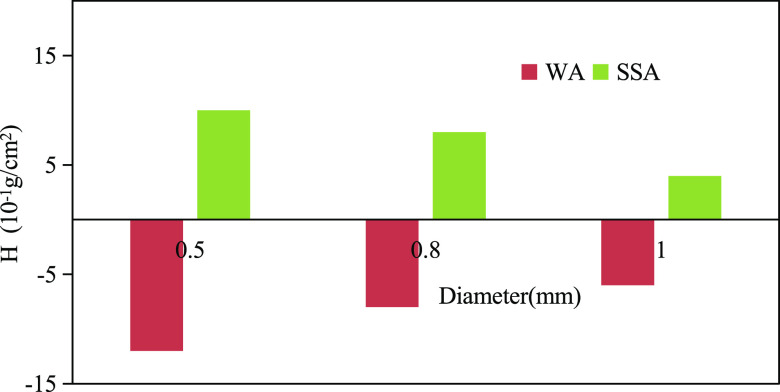
Silicone capillary water wettability.

As shown in Figure 2, under WA conditions, the *H* in the silicone capillary is a negative value, so relative to the air, water is the nonwet phase. After the surfactant was added, the *H* changes from a negative value to a positive value. Meanwhile, relative to the air, the water changes from a nonwet phase to a wet phase.

**Figure 3:**
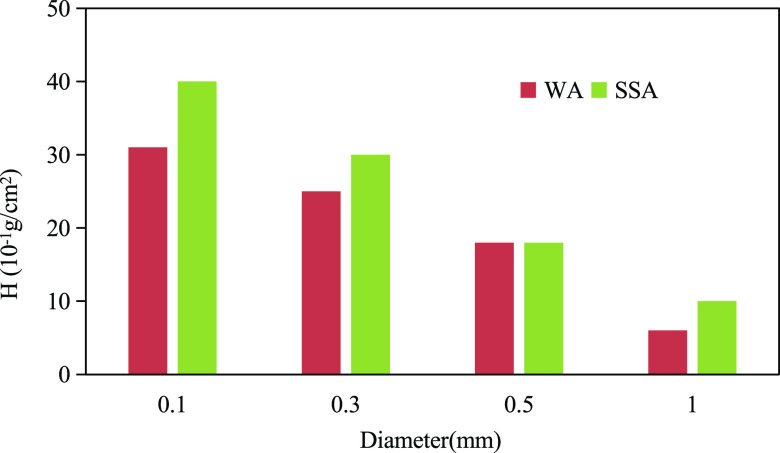
Water wettability of the quartz capillary treated with mineral oil.

As shown in Figure 3, under WA conditions, the *H* in the quartz capillary treated with mineral oil is a positive value, so relative to the air, water is the wet phase. After the surfactant was added, the value of *H* and the capillary pressure increase. The addition of surfactants improves the water wettability of the capillary and reduces the interfacial tension. The former increases the capillary pressure and the latter reduces the capillary pressure. We believe that the former has a greater impact on the capillary pressure than the latter, so the capillary pressure increases. Comparing to Figure 1, we found that the effect of a surfactant on the water wettability of the capillary treated with mineral oil is better than that of the untreated capillary, which means that the surfactant can not significantly improve the water wettability of strong water wetting capillary.

Comparing the values of *H* under the same conditions as shown in Figures 1–3, we can see that relative to the air, the water wettability from strong to weak is as follows quartz capillary > oil-treated quartz capillary > silicone capillary.

### 2.2 Spontaneous Imbibition of the Untreated Quartz Capillary

Capillary action refers to the wetting of the solid surface by the liquid due to the difference in the cohesion of the liquid and the adhesion between the liquid and the solid when the liquid is in contact with the tube wall. The shape of the two-phase meniscus in the capillary, the radius of the curvature of the meniscus, and the size of the two-phase interfacial tension directly determine the size and the direction of the capillary pressure.

As shown in Table 1, under the condition of WA and an equal pipe diameter, the menisci do not move in the capillaries with radii of 50 and 25, while the menisci in the capillaries with radii of 15 and 5 move and bubbles are continuously expelled. Under the condition of an equal pipe diameter and SSA, all menisci move and bubbles are continuously discharged. Under the condition of a nonequal pipe diameter, all menisci move from the small pipe diameter end to the large pipe diameter end, and bubbles are continuously discharged, which is consistent with the results of previous studies.^24,25^

**Table 1:** Behavior of the Meniscus in Horizontal Quartz Capillaries^a^

WA			SSA		
radius/10 μm			radius/10 μm		
left	right	meniscus behavior	left	right	meniscus behavior
50	50	static	50	50	move and bubbles are expelled
25	25	static	25	25	move and bubbles are expelled
15	15	move and bubbles are expelled	15	15	move and bubbles are expelled
5	5	move and bubbles are expelled	5	5	move and bubbles are expelled
50	25	move from right to left and bubbles are expelled	50	25	move from right to left and bubbles are expelled
15	5	move from right to left and bubbles are expelled	50	15	move from right to left and bubbles are expelled
50	15	move from right to left and bubbles are expelled	50	5	move from right to left and bubbles are expelled

a Note: The static of the meniscus means that there is no meniscus in the capillary, and the air fills the entire capillary, or the meniscus has a certain displacement in the capillary due to the instant high-frequency vibration of the capillary, but there is no continuous bubble discharge behavior.

**Figure 4:**
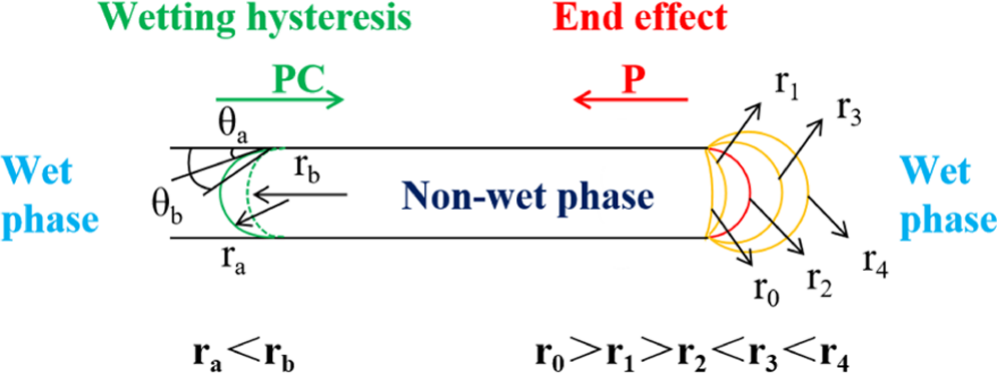
Behavior of menisci at both ends of the nonwet phase in the equal diameter capillary.

Some researchers found that the wetting angle at the meniscus of the entry end was not constant using different liquids to test the spontaneous imbibition process of the capillary. During the movement of the meniscus, the dynamic contact angle is greater than the static contact angle and is related to the self-absorption speed of the fluid.^29−31^ This is a wetting hysteresis phenomenon.^32−35^ The capillary end effect^26,27,36^ refers to an additional resistance phenomenon caused by the deformation of the meniscus when the capillary suddenly loses continuity, and the nonwet phase leaves the capillary port to enter the wet phase. As shown in Figure 4, the capillary pressure (PC) and the additional pressure (*P*) are generated by the menisci at both ends of the air phase in the capillary tube, and the magnitude and the direction of the pressures are the key factors for the movement of the meniscus and expulsion of bubbles. For a horizontally placed capillary, the hydrostatic pressures at two ports are equal and opposite, and if the fluid viscosity is neglected, the fluid system in the capillary is only affected by the curved surface pressure difference generated by the menisci at both ends of the air phase.

**Figure 5:**
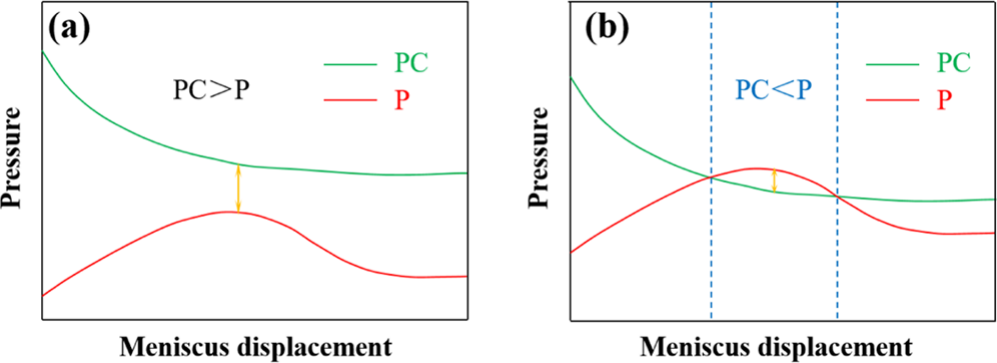
Variation of meniscus pressures at both ends of the nonwet phase in the equal diameter capillary. (a) Pressure difference is always positive and (b) there is a negative pressure difference interval.

For the process of moving the meniscus in the equal diameter capillary, we believe that the wetting hysteresis will cause the dynamic contact angle of the meniscus at the inlet end to increase first and then fluctuate around a certain value, which means that the capillary pressure PC is reduced to a certain value and then levels out. However, the radius of the curvature of the meniscus at the bubble discharge end of the capillary decreases first and then increases, which causes the additional pressure *P* to increase first and then decrease, as shown in Figure 4. During the entire period from the bubble generation to the bubble leaving the capillary, the additional pressures of the meniscus at both ends of the nonwet phase change with the displacement of the meniscus at the liquid inlet end, as shown in Figure 5. When the pressure difference value is always positive, as shown in Figure 5a, the meniscus keeps moving and bubbles are continuously discharged. When the pressure difference appears negative, as shown in Figure 5b, and if the meniscus moves under the action of inertia and crosses the negative interval, the meniscus can keep moving and bubbles are continuously discharged, but if the movement speed of the meniscus is reduced to 0 in the negative interval, the fluid system inside the capillary reaches a force balance under the dual adjustment of the end effect and the wetting hysteresis, and the meniscus remains stationary and the capillary stops expelling bubbles.
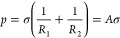
2where *R*_1_ and *R*_2_ are the parameters describing the curvature radius of the meniscus, σ is the interfacial tension of the two phases, and *A* is the parameter related to the curvature radius. The larger the radius of the curvature, the smaller the value of *A*.

**Figure 6:**
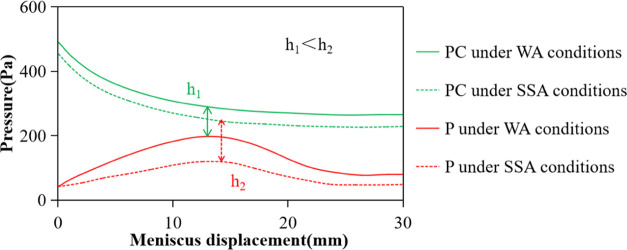
Influence of the surfactant on meniscus pressures in the strong water wetting capillary: *r* = 25 × 10 μm^2^.

**Figure 7:**
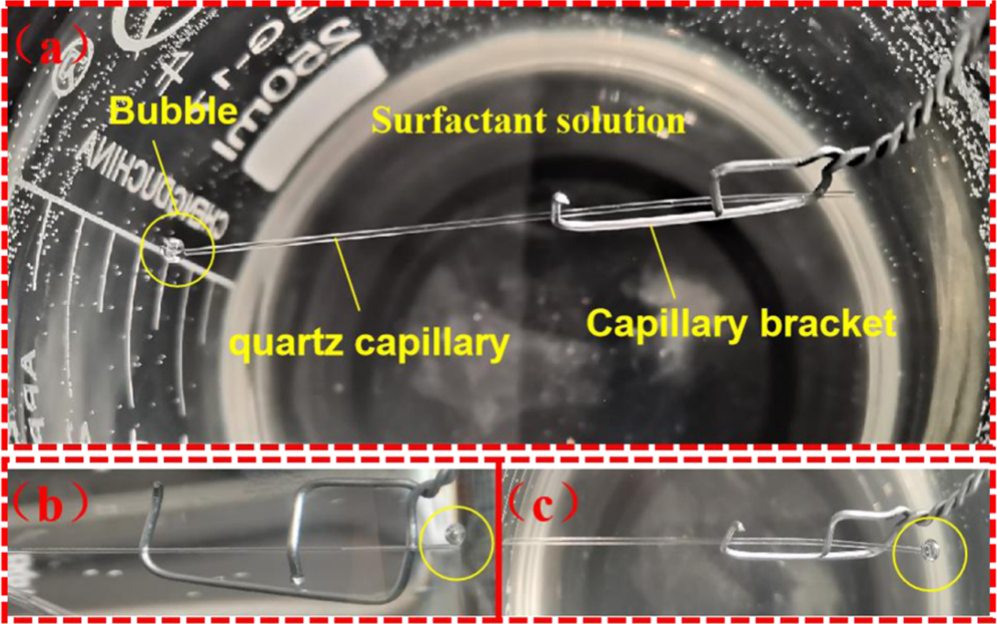
Bubbles are continuously discharged: (a) phenomenon of the first experiment, (b) front view of the phenomenon in the second experiment, and (c) top view of the phenomenon in the second experiment.

The additional pressure *P* produced by the end effect of a single capillary in the process of imbibition is opposite to the capillary force PC, and the *P* is the resistance in the process of imbibition. The *P* on the curved surface can be described by the Laplace equation (eq 2). The addition of surfactants reduces the interfacial tension σ, so from the Laplace equation (eq 2), it can be seen that the additional pressure *P* and the change range of *P* at the bubble discharge end reduce compared to WA conditions. Moreover, as shown in Figure 1 above, the addition of surfactants also reduces the capillary pressure PC compared to WA conditions. As shown in Table 1, compared with WA conditions, the addition of surfactants causes the menisci in the capillaries with radii of 50 and 25 to move, and bubbles are continuously discharged. According to the analysis, the addition of surfactants causes the decrease in PC at the liquid inlet end to be lower than the decrease in the additional pressure *P* at the bubble discharge end, which increases the pressure difference between the two ends, as shown in Figure 6. Therefore, under the condition of an equal diameter capillary and WA, the addition of surfactants is conducive to the occurrence of spontaneous imbibition, as shown in Figure 7.

**Figure 8:**
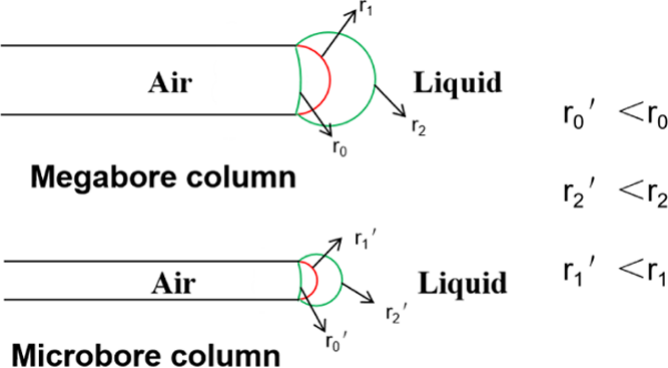
Influence of tube diameter reduction on the meniscus curvature radius at the capillary bubble discharge end.

**Figure 9:**
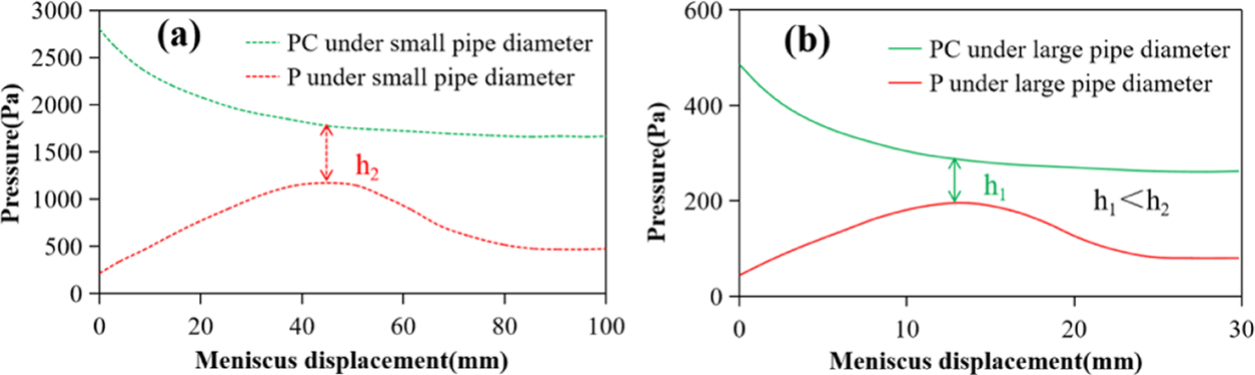
Influence of pipe diameter reduction on the meniscus pressures on both sides of the nonwet phase: (a) *r* = 5 × 10 μm^2^ and (b) *r* = 25 × 10 μm^2^.

When the diameter of the capillary decreases, the capillary pressure PC at the liquid inlet increases, and the additional pressure *P* at the bubble discharge end also increases due to the decrease in the radius of the curvature, as shown in Figure 8. As shown in Table 1, under WA conditions, the meniscus moves, and bubbles are continuously discharged after the capillary diameter decreases. We analyze that with the decrease of the capillary diameter, the increase of the capillary pressure PC at the liquid inlet end is higher than the increase of the additional pressure *P* at the bubble discharge end, which also increases the pressure difference between the two ends, as shown in Figure 9. Therefore, the reduction of the capillary diameter is conducive to the occurrence of spontaneous imbibition.

### 2.3 Spontaneous Imbibition of the Treated Quartz Capillary

As shown in Table 2, compared with Table 1, the water wettability of the treated quartz capillaries is reduced, and the menisci still move from the small diameter end to the large diameter end, while the menisci in the equal diameter capillary no longer move.

**Table 2:** Meniscus Behavior of the Treated Quartz Capillary at the Horizontal Level

radius/10 μm			
left	right	meniscus behavior in the condition of WA	meniscus behavior in the condition of SSA
5	5	static	static
15	15	static	static
25	25	static	static
50	50	static	static
50	25	move from right to left and bubbles are discharged	move from right to left and bubbles are discharged
50	15	move from right to left and bubbles are discharged	move from right to left and bubbles are discharged
50	5	move from right to left and bubbles are discharged	move from right to left and bubbles are discharged
25	5	move from right to left and bubbles are discharged	move from right to left and bubbles are discharged

**Figure 10:**
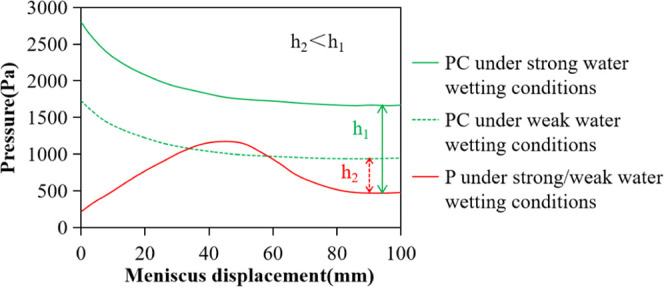
Influence of the decrease in water wetness on the meniscus pressures in the capillary: *r* = 5 × 10 μm^2^.

Under the condition of an equal pipe diameter, although a small amount of the wetting phase invaded at one end of the capillary to establish the initial state of spontaneous imbibition under the action of high-frequency vibration, the decrease of the capillary water wettability causes the capillary pressure PC to decrease at the liquid inlet end, and the additional pressure *P* at the bubble discharge end remains unchanged, so the pressure difference at both ends decreases, as shown in Figure 10. The viscous force inside the liquid is easy to balance the pressure difference so that the fluid system in the capillary tends to be force-balanced, which makes the meniscus stand still.

Under the condition of an equal pipe diameter and SSA, although the addition of a surfactant improves the water wettability of the treated quartz capillary, comparing the value of *H* in the same pipe diameter in Figures 1 and 3, it is found that under weak water wetting conditions, the addition of surfactants cannot greatly increase the capillary pressure PC at the liquid inlet end. Therefore, the increase in the pressure difference between the two ends is limited, and it is not enough to move the meniscus. Therefore, improving the water wettability by surfactants may not achieve favorable spontaneous imbibition, and the initial water wet strength of the capillary is the key to the occurrence of spontaneous imbibition. However, pieces of literature related to oil production by imbibition show that a surfactant solution can significantly improve the effect of spontaneous imbibition under suitable interfacial tension conditions (mostly 10^–1^ mN/m), which make the efficiency of spontaneous imbibition reach a higher level.^37,38^ According to analysis, different surfactants have different abilities to improve the spontaneous imbibition of capillaries with different water wettabilities. The addition of the surfactant can reduce the wetting angle θ and interfacial tension σ at the same time, but the change of the capillary pressure is opposite. The initial water wet strength of the capillary and the properties of the surfactant directly determine the change of the capillary pressure, which, in turn, affects the effect of spontaneous imbibition.

### 2.4 Spontaneous Imbibition of the Quartz Capillary with One End Treated

**Figure 11:**
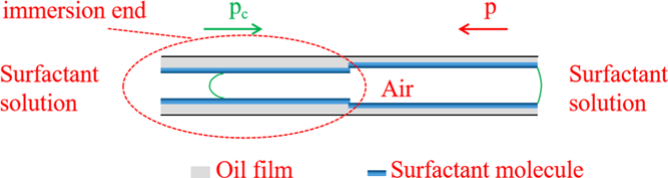
Schematic diagram of spontaneous imbibition of the quartz capillary treated at one end.

In the process of mineral oil processing the quartz capillary, one end of the quartz capillary is immersed in the mineral oil, and the oil is instantly adsorbed on the inner wall surface of the whole capillary in the form of a film.^39−41^ Due to the internal viscous force of the liquid, even if the oil is blown out from the other end of the capillary, the thickness of the oil film adhering to the inner wall of the capillary at the immersion end is still greater than that at the other end, as shown in Figure 11.

As shown in Table 3, the menisci in the equal diameter capillary do not move under WA conditions. After the surfactant was added, the menisci in the capillaries move from the end with the thicker oil film to the end with the thinner oil film, and bubbles are continuously discharged. It is analyzed that under the condition of WA, due to the reduced water wettability of the capillary, the capillary pressure PC at the inlet end is small, and the pressure difference between the two ends is not enough to push the meniscus to move. After the surfactant is added, the active agent molecules diffuse and adsorb on the inner wall of the capillary, which improves the water wettability of the capillary and increases the capillary pressure PC at the inlet end. In addition, the actual flow cross-sectional radius of the fluid at the thicker oil film end is smaller than that at the other end, which further increases the capillary pressure PC. But the actual flow cross-sectional radius of the fluid at the end of the thinner oil film is larger than that at the other end, which makes the additional pressure *P* at the end of the capillary outlet smaller. The pressure difference between the two ends is increased to the extent that it is sufficient to push the meniscus to move, so the meniscus moves from the thick oil film end to the thin oil film end, and bubbles are continuously discharged. The thickness of the nonwet phase on the inner wall of the equal diameter capillary affects the direction in which the wet phase is immersed.

**Table 3:** Behavior of the Meniscus in the Quartz Capillary with One End Treated

radius/10 μm			
left (thick hydrophobic layer)	right (thin hydrophobic layer)	meniscus behavior in the condition of WA	meniscus behavior in the condition of SSA
5	5	static	move from left to right and bubbles are discharged
15	15	static	move from left to right and bubbles are discharged
25	25	static	move from left to right and bubbles are discharged
50	50	static	move from left to right and bubbles are discharged

### 2.5 Spontaneous Imbibition of Capillaries with Different Water Wettabilities at Both Ends

As shown in Table 4, under SSA conditions, all of the menisci move from the quartz end to the silicone end, and bubbles are continuously discharged. Although the diameter of the silicone end is smaller than that of the quartz end, there is no phenomenon that the meniscus moves from the silicone end to the quartz end.

**Table 4:** Behavior of Menisci in Capillaries with Different Water Wettabilities at Both Ends

radius/10 μm		
left (quartz end)	right (silicone end)	meniscus behavior in the condition of SSA
50	50	move from left to right and bubbles are discharged
50	40	move from left to right and bubbles are discharged
25	25	move from left to right and bubbles are discharged
15	25	move from left to right and bubbles are discharged

As shown in Figures 1 and 2 above, although the addition of a surfactant reduces the capillary pressure of the quartz capillary and increases the capillary pressure of the silicone capillary, comparing the value of *H* in the same diameter of the quartz capillary and the silicone capillary under SSA conditions, it is found that the range of reduction and increase is limited, and the capillary force of the former is significantly greater than that of the latter. It is analyzed that even if the water wettability of the silicone capillary is improved by surfactants, only when the quartz end is used as the entry end, the pressure difference is sufficient to move the meniscus, and bubbles are discharged. It is further illustrated that the initial water wet strength of the capillary is the key to the occurrence of spontaneous imbibition.

### 2.6 Comment on the Limitations of the Experiments and Plans for Future Work

Spontaneous imbibition is an effective way to develop tight oil/gas reservoirs. Liquid can penetrate into the reservoir through self-absorption. Therefore, an in-depth study of the capillary imbibition behavior is of great significance for understanding the spontaneous imbibition mechanism of displacement fluid in the bedrock. In actual low-permeability tight oil/gas layers, the pores and fractures are mostly nanomicron in size,^16^ and the physical and chemical properties are complex and changeable. The wetting hysteresis and end effects are influenced by many factors, such as the roughness of the pore throat surface, the cross-sectional shape of the pore throat, the composition of the adsorption layer on the pore throat surface,^28^ and nonuniform wetting of the pore throat. To improve the application effect of imbibition oil recovery in the development of tight oil/gas reservoirs, we need to combine a large number of actual oil reservoir imbibition oil production data, statistically distinguish the main macrocontrol factors that affect imbibition oil production, and further subdivide oil and gas reservoir categories. In addition, the effect of the increase of the dynamic contact angle and its fluctuation range due to the wetting lag in the spontaneous imbibition of the capillary needs to be further explored.

## 3 Conclusions

Through the abovementioned experiments and discussion, the following conclusions can be drawn within the capillary size range of the order of 10–100 μm.

(1) When the capillary diameter and two-phase fluid are unchanged, it is good that the method of measuring the converted height of the self-absorbent phase is used to determine the strength of the capillary water wettability.
(2) In strong water wetting equal diameter capillaries, the addition of surfactants or the reduction of the capillary diameter is conducive to the occurrence of spontaneous imbibition.
(3) In weak water wetting equal diameter capillaries, the addition of a surfactant or the change of the capillary diameter has little effect on spontaneous imbibition.
(4) In the equal diameter capillary, the addition of a surfactant is easy to make the end with the thicker hydrophobic adhesion layer on the inner wall of the capillary become the entrance end of the meniscus.
(5) In terms of surfactants to improve wettability, weakly wetted materials are better than strong wetted materials. However, the strong initial water wettability of the capillary plays a more important role in spontaneous imbibition.
(6) When surfactants are used to change the water wettability of materials to improve spontaneous imbibition efficiency, the initial water wet strength of the capillary and the comprehensive changes in the capillary pressure caused by interfacial tension need to be considered.

## 4 Materials and Methods

### 4.1 Materials

The main component of mineral oil is C_16_–C_31_ normal alkanes and isoparaffins, the relative molecular mass is in the range of 300–400, the surface tension is 23 mN/m, and the apparent viscosity is 20 MPa·s at atmospheric pressure and 25 °C. The water quality analysis of experimental water is shown in Table 5. The surface tension of the water is 72.1 mN/m at 25 °C and atmospheric pressure. A BHS-01A anionic surfactant has good interfacial activity, it can reduce the oil–water interfacial tension to 10^–3^ mN/m and change the water wettability of quartz and silicone. The material of the quartz capillary is uniform, and the circulation radii are 50, 150, 250, and 500 μm, respectively. The material of the silicone capillary is uniform too, and the flow radii are 250, 400, and 500 μm, respectively. The silicone capillary is an organic silica capillary, which is like a rubber capillary, and the quartz capillary is a glass capillary. The wettability of the two is quite different.

**Table 5:** Analysis of the Clear Water Quality

	HCO_3_^–^ (mg/L)	Ca^2+^ (mg/L)	Mg^2+^ (mg/L)	Cl^–^ (mg/L)	SO_4_^2–^ (mg/L)	K^+^ + Na^+^ (mg/L)	total salinity (mg/L)
salinity	101.1	75.6	4.2	147.1	0.7	123.1	452.0

### 4.2 Experimental Methods

#### 4.2.1 Capillary Water Wettability Measurement

**Figure 12:**
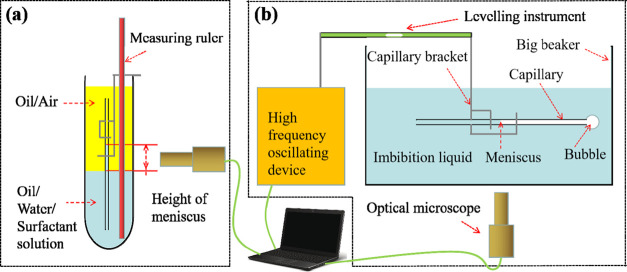
Schematic diagrams of experiments: (a) schematic diagram of the capillary water wettability performance test and (b) schematic diagram of the capillary spontaneous imbibition experiment.

Quartz capillary tubes and silicone capillary tubes were individually placed on the capillary bracket and then vertically placed on the water–air interface (WA) and the surfactant solution air interface (SSA). The interface behavior in the capillary was observed through an optical microscope for a long time until the interface state was stable, and the height of the meniscus was recorded, as shown in Figure 12a. The measurements were repeated three times and the average value was recorded.

#### 4.2.2 Mineral Oil-Treated Quartz Capillary

Some quartz capillaries with different diameters were totally immersed in mineral oil to discharge the bubbles. The capillaries were soaked for 48 h and then taken out. A rubber suction ball was used to blow out the oil in the capillaries, and then the capillaries were placed in a fume hood to dry for later use.

#### 4.2.3 One End of the Quartz Capillary Treated with Mineral Oil

One end of quartz capillaries with different diameters was put in mineral oil upright for 48 h and then taken out. A rubber suction ball was used to blow out the oil in the capillaries from the nonoil-immersed end, and then the capillaries were placed in a fume hood to dry for later use.

#### 4.2.4 Spontaneous Imbibition of Equal Diameter Capillaries

Different quartz capillaries and silicone capillaries were put on the capillary bracket individually. The capillary tubes were quickly placed horizontally in clean water or a 0.3% (wt) BHS-01A solution through the bracket. The instantaneous high-frequency vibration of the bracket was controlled so that the wetting phase was immersed into one end of the capillary, which is the initial state of spontaneous imbibition. The bubble discharge behavior of the capillary was observed through an optical microscope, and the additional pressure values at different displacements were calculated using the shape of the meniscus, as shown in Figure 12b. The experiment was repeated three consecutive times until the behavior was the same.

#### 4.2.5 Spontaneous Imbibition of Unequal Diameter Capillaries

Capillary ports of different pipe diameters were connected, and the capillaries were made in the same straight line. The joint was sealed with petroleum jelly to make an unequal diameter capillary. The experiment method of spontaneous imbibition is the same as the one described above (Section 4.2.4).
